# Identification of mutation patterns and circulating tumour DNA-derived prognostic markers in advanced breast cancer patients

**DOI:** 10.1186/s12967-022-03421-8

**Published:** 2022-05-13

**Authors:** Hao Liao, Jiayang Zhang, Tiantian Zheng, Xiaoran Liu, Jianxin Zhong, Bin Shao, Xiaoxi Dong, Xiaohong Wang, Pan Du, Bonnie L. King, Shidong Jia, Jianjun Yu, Huiping Li

**Affiliations:** 1 Key Laboratory of Carcinogenesis and Translational Research (Ministry of Education/Beijing), Department of Breast Oncology, Peking University Cancer Hospital & Institute, 52 Fucheng Rd, Beijing, 100142 China; 2 Huidu Shanghai Medical Sciences Ltd, Shanghai, 201499 China

**Keywords:** Next generation sequencing, Advanced breast cancer, ctDNA fraction, Tumor mutation burden, Survival outcomes

## Abstract

**Background:**

The correlations between circulating tumour DNA (ctDNA)-derived genomic markers and treatment response and survival outcome in Chinese patients with advanced breast cancer (ABC) have not been extensively characterized.

**Methods:**

Blood samples from 141 ABC patients who underwent first-line standard treatment in Peking University Cancer Hospital were collected. A next-generation sequencing based liquid biopsy assay (PredicineCARE) was used to detect somatic mutations and copy number variations (CNVs) in ctDNA. A subset of matched blood samples and tumour tissue biopsies were compared to evaluate the concordance.

**Results:**

Overall, TP53 (44.0%) and PIK3CA (28.4%) were the top two altered genes. Frequent CNVs included amplifications of ERBB2 (24.8%) and FGFR1 (8.5%) and deletions of CDKN2A (3.5%). PIK3CA/TP53 and FGFR1/2/3 variants were associated with drug resistance in hormone receptor-positive (HR +) and human epidermal growth factor receptor 2-positive (HER2 +) patients. The comparison of genomic variants across matched tumour tissue and ctDNA samples revealed a moderate to high concordance that was gene dependent. Triple-negative breast cancer (TNBC) patients harbouring TP53 or PIK3CA alterations had a shorter overall survival than those without corresponding mutations (*P* = 0.03 and 0.008). A high ctDNA fraction was correlated with a shorter progression-free survival (PFS) (*P* = 0.005) in TNBC patients. High blood-based tumor mutation burden (bTMB) was associated with a shorter PFS for HER2 + and TNBC patients (*P* = 0.009 and 0.05). Moreover, disease monitoring revealed several acquired genomic variants such as ESR1 mutations, CDKN2A deletions, and FGFR1 amplifications.

**Conclusions:**

This study revealed the molecular profiles of Chinese patients with ABC and the clinical validity of ctDNA-derived markers, including the ctDNA fraction and bTMB, for predicting treatment response, prognosis, and disease progression.

*Trial registration*: ClinicalTrials.gov ID: NCT03792529. Registered January 3rd 2019, https://clinicaltrials.gov/ct2/show/NCT03792529.

**Supplementary Information:**

The online version contains supplementary material available at 10.1186/s12967-022-03421-8.

## Introduction

Female breast cancer has become the most commonly diagnosed malignancy worldwide according to global cancer statistics, with nearly 2.3 million new cases in 2020 [1]. Compared with other malignancies, such as lung cancer, patients with breast cancer have relatively favourable treatment response rates and therefore longer 5-year survival rates (89.7%, by SEER dataset) [2]. However, a significant percentage of breast cancer patients eventually develop incurable metastatic disease, which is characterized by an increasingly complex genomic landscape [3]. Traditional Sanger sequencing cannot fulfil the requirements of rapidly generating comprehensive correlations between genomic variants and treatment response in a large number of clinical samples [4]. Therefore, robust genomic profiling technologies should be applied to the characterization of advanced breast cancer (ABC) with the goal of defining genomic variation, evaluating prognosis and developing biomarkers to guide treatment selection.

Next generation sequencing (NGS) and molecular testing assays have evolved rapidly in recent years and are being successfully adopted in various clinical applications. When high coverage is achieved, targeted NGS enables the detection of low-frequency somatic mutations in heterogeneous tumour populations and circulating cell-free DNA (cfDNA) [5]. A number of gene mutations, such as those in ESR1, PIK3CA, and TP53, have been found to be associated with drug resistance and worse prognosis in breast cancer [6]. Given the strong detection capacity of NGS, it is now possible for clinicians to identify certain resistance-related mutations in a timely manner and make new therapeutic choices [7]. Despite remaining challenges and limitations, efficient high-throughput sequencing technologies are leading to real and unprecedented benefits for the medical care of cancer patients [8].

Circulating tumour DNA (ctDNA) is the small proportion of cfDNA that is released into the circulation from tumour cells and is detectable in a wide range of malignancies [9]. Several studies have demonstrated high concordance between alterations detected in ctDNA and tumour tissue biopsy in ABC [10–14]. Moreover, ctDNA can serve as a noninvasive biomarker for disease monitoring, predicting drug efficacy, and determining prognosis [15]. The ctDNA fraction is defined as the proportion of ctDNA in cfDNA. In other types of cancer, such as metastatic prostate cancer, the ctDNA fraction has been shown to be a prognostic factor [16]. Blood-based tumour mutation burden (bTMB) is a metric that represents the total number of mutations per coding area of a tumour genome, and is a metric that is frequently generated by NGS analysis [17]. The magnitude and prognostic impact of bTMB is significantly different across solid tumour types [18]. In triple-negative breast cancer (TNBC), bTMB is an emerging prognostic biomarker for immunotherapy [19, 20]. However, it remains unclear in ABC patients whether the ctDNA fraction and bTMB correlate with different subtypes, treatment response or prognosis.

Herein, we investigated the molecular profiles of samples from Chinese ABC patient with a targeted 152-gene NGS panel. A comparison of the mutation profiles across matched tumour tissue and plasma ctDNA samples was conducted. Then, several genomic aberrations related to the treatment response were identified. Moreover, we explored whether the ctDNA fraction and bTMB could be used to evaluate treatment response and prognosis, respectively. Finally, we evaluated the clinical validity of ctDNA sequencing for longitudinally monitoring disease progression.

## Materials and methods

### Patient cohort and clinical data collection

The study protocol strictly followed the Declaration of Helsinki and was approved by the Peking Universities Cancer Hospital Ethics Committee (CABC008, 2020KT75). All patients signed a written informed consent form prior to registration for ctDNA-analysis. Female patients with first-line metastatic or primary stage IV disease who were diagnosed at Peking University Cancer Hospital (PKUCH) were enrolled in this prospective study from December 2012 to June 2021.

The clinical data collected in this study included receptor status (oestrogen receptor (ER), progesterone receptor (PR), and human epidermal growth factor receptor 2 (HER2)), age, tumour grade, Ki67, primary TNM stage, adjuvant treatment, progression free-survival (PFS), metastasis site, disease status, and treatment response. The evaluation of the receptor status by immunohistochemistry (IHC) was based on the American Society of Clinical Oncology guidelines [21, 22]. A specimen with a minimum of 1% invasive tumour cells positive for ER/PR was considered hormone receptor-positive (HR +). HER2 positivity was defined as IHC staining 3 + or 2 + with gene amplification assessed by fluorescence in situ hybridization (FISH) with a HER2/CEP17 ratio ≥ 2.0 and average HER2 copy number ≥ 4.0 signals/cell. TNM staging was defined according to the American Joint Committee on Cancer staging manual for breast cancer [23]. All patients were treated with the current standard therapy defined by the NCCN clinical practice guidelines [24]. The treatment response was evaluated according to RECIST 1.1 every 2–3 months of treatment duration. When a patient’s first-line PFS was less than 3 months, the patient was designated as having drug resistance. Clinical information was collected by reviewing electronic medical records, and survival data were recorded using a follow-up database in PKUCH.

### Sample collection

Baseline plasma samples were collected from all 141 patients to identify the mutation pattern of ABC. Metastatic tumour biopsies were obtained from 21 of the 141 patients to validate the concordance between plasma and tumour tissue. Moreover, extra plasma samples were collected from 31 of the 141 patients to dynamically monitor the disease. Blood samples (5–10 ml) were collected into EDTA-containing blood collection tubes and processed within 2 h of collection by centrifugation at 1600*g* for 20 min at room temperature. Plasma was separated from buffy coats and red blood cells, aliquoted, and stored at − 80 °C until ctDNA extraction.

### ctDNA extraction, library construction, and sequencing

A highly sensitive NGS assay (PredicineCARE, a commercially available 152-gene cancer NGS panel, developed by Huidu Shanghai Medical Sciences Ltd.) was used to detect somatic mutations and copy number variations (CNVs) in ctDNA. The ctDNA was extracted from 1 to 2 ml of the isolated plasma samples using the QIAamp Circulating Nucleic Acid Kit (QIAamp, Venlo, NL). Then, the quantity and quality of the purified ctDNA were checked using the Qubit 3.0 Fluorometer (Thermo Fisher Scientific, Waltham, MA, USA) and Bioanalyzer 2100 (Agilent Technologies, Santa Clara, CA, USA). A total of 5–30 ng of purified ctDNA was subjected to library construction, including end-repair dA-tailing and adapter ligation. Ligated library fragments with appropriate adapters were amplified via PCR. The amplified DNA libraries were then checked using the Bioanalyzer 2100. All the samples in this study had a yield > 700 ng and were processed for hybrid capture.

Library capture was conducted using biotin-labelled DNA probes (Thermo Fisher Scientific, Weltham, MA, USA). In brief, the library was hybridized using the PredicineCARE panel overnight and captured with Dynabeads M-270 Streptavidin (Thermo Fisher Scientific, Weltham, MA, USA). The unbound fragments were washed away, and the enriched fragments were amplified via PCR amplification. For library preparation, the purified product was assessed using the Bioanalyzer 2100 and loaded into the NextSeq 500 (Illumina, San Diego, CA, USA) for NGS with paired-end 2 × 150 bp sequencing kits. The average sequencing depth per sample was approximately 20,000 × before deduplication. NGS quality-assessment was performed by examining the percentage of targeted regions with sufficient unique coverage (greater than 1500x). Samples with > 80% regions having > 1500 × unique coverage were deemed to pass Quality Control and were included in this study.

### Sequencing data analysis

Sequencing data were analysed using an in-house-developed NGS analysis pipeline developed by Huidu Shanghai Medical Sciences Ltd., as described in previous studies [16, 25]. This process begins with the analysis of the raw sequencing data (BCL files) and outputs the final mutation calls. First, the pipeline performed adaptor trimming, barcode checking, and correction. Cleaned paired FASTQ files were aligned to the human reference genome build hg19 using the Burrows–Wheeler Aligner alignment tool (version 0.7.15). Then, consensus bam files were derived by merging paired-end reads originating from the same molecules (based on mapping location and unique molecular identifiers) as single-strand fragments. Single-strand fragments from the same double stranded DNA molecules were merged to be double-stranded to reduce sequencing and PCR errors during this process. Detected variants were filtered based on the local variant background (defined by plasma samples from healthy Chinese donors and internal sample pools), log-odds threshold [26], base quality and mapping quality thresholds, and repeat regions. Variants with mutation allele frequency (MAF) ≥ 0.25% and hotspot variants with MAF ≥ 0.1% were reported. Benign and likely benign variants were excluded.

### Statistical analysis

Categorical variables are described as frequencies (percentage), and continuous variables are presented as medians (range). Fisher’s exact test was used to evaluate differences in the rate of mutations across specific genes in different molecular subtypes. Survival analysis was used to evaluate the associations between ctDNA-based variables measured prior to the commencement of treatment and PFS and overall survival (OS). Associations between the variables and PFS and OS were estimated by plotting Kaplan–Meier curves and compared by log-rank test to determine significant differences among subgroups. A univariate Cox regression model was performed to compute medians and 95% confidence intervals (CIs) for prognostic variables. The Wilcoxon test or Kruskal–Wallis test was used to compare the ctDNA fraction and bTMB between different molecular subtypes. To dichotomize the variables for the survival analysis, the upper quartile cut-off was used for both the ctDNA fraction and bTMB. Cohen's kappa was used to evaluate the concordance of variants between plasma and tissue samples. All statistical analyses were performed using R language, version 3.5.3. A two-sided *P value* of < 0.05 was considered significant.

## Results

### Baseline characteristics of patients

In total, 141 ABC patients who underwent systematic treatment at PKUCH from December 2012 to June 2021 were included in this study. All patients were female. The median age was 48.0 years, and the age range was 21.0 to 77.0 years (Table 1). Among these patients, the proportions of patients with HR + , human epidermal growth factor receptor 2-positive (HER2 +), and triple-negative disease were 31.2% (44/141), 41.1% (58/141), and 27.7% (39/141), respectively. As shown in Table 2, all patients developed metastatic disease, and the metastatic sites included the chest wall, bone, lymph nodes, lung, liver, brain and others sites. More than half of these patients had visceral metastasis (52.5%, 74/141) and 71.0% (100/141) had ≥ 2 sites of metastasis. Current standard therapy was applied to all patients once metastatic/advanced disease was diagnosed. With respect to adjuvant therapy, 72.3% (102/141) of all patients had received chemotherapy. In the HR + patient subgroup, 62.1% (36/58) had received adjuvant endocrine therapy.

**Table 1:** The characteristics of 141 advanced breast cancer patients in baseline samples

	HR + (N = 58)	HER2 + (N = 44)	TN (N = 39)	Overall (N = 141)
Age, median (range)	46.0 (29.0, 77.0)	52.0 (27.0, 76.0)	48.0 (21.0, 73.0)	48.0 (21.0, 77.0)
Tumour grade				
I	2 (3.4%)	1 (2.3%)	1 (2.6%)	4 (2.8%)
II	41 (70.7%)	28 (63.6%)	16 (41.0%)	85 (60.3%)
III	8 (13.8%)	9 (20.5%)	18 (46.2%)	35 (24.8%)
Unknown	7 (12.1%)	6 (13.6%)	4 (10.2%)	17 (12.1%)
Ki67 (%)				
1–14	10 (17.2%)	1 (2.3%)	3 (7.7%)	14 (9.9%)
15–25	12 (20.7%)	8 (18.2%)	5 (12.8%)	25 (17.7%)
26–50	19 (32.8%)	19 (43.2%)	13 (33.3%)	51 (36.2%)
> 50	11 (19.0%)	13 (29.5%)	10 (25.6%)	34 (24.1%)
Unknown	6 (10.3%)	3 (6.8%)	8 (20.5%)	17 (12.1%)
Primary TNM stage				
I	6 (10.3%)	3 (6.8%)	9 (23.1%)	18 (12.8%)
II	19 (32.8%)	12 (27.3%)	13 (33.3%)	44 (31.2%)
III	17 (29.3%)	12 (27.3%)	8 (20.5%)	37 (26.2%)
IV	13 (22.4%)	13 (29.5%)	8 (20.5%)	34 (24.1%)
Unknown	3 (5.2%)	4 (9.1%)	1 (2.6%)	8 (5.7%)
Adjuvant CT				
No	3 (5.2%)	3 (6.8%)	0 (0%)	6 (4.3%)
Primary IV	9 (15.5%)	12 (27.3%)	7 (17.9%)	28 (19.9%)
E-based	13 (22.4%)	4 (9.1%)	6 (15.4%)	23 (16.3%)
T-based	4 (6.9%)	5 (11.4%)	7 (17.9%)	16 (11.3%)
E + T	28 (48.3%)	20 (45.5%)	15 (38.5%)	63 (44.7%)
Unknown	1 (1.7%)	0 (0%)	4 (10.3%)	5 (3.5%)
Adjuvant ET				
No	19 (32.8%)	28 (63.6%)	30 (76.9%)	77 (54.6%)
SERM	21 (36.2%)	7 (15.9%)	3 (7.7%)	31 (22.0%)
AI	10 (17.2%)	9 (20.5%)	2 (5.1%)	21 (14.9%)
SERM + AI	5 (8.6%)	0 (0%)	0 (0%)	5 (3.5%)
Unknown	3 (5.2%)	0 (0%)	4 (10.3%)	7 (5.0%)
Adjuvant RT				
No	37 (63.8%)	29 (65.9%)	24 (61.5%)	90 (63.8%)
Yes	21 (36.2%)	15 (34.1%)	11 (28.2%)	47 (33.3%)
Unknown	0 (0%)	0 (0%)	4 (10.3%)	4 (2.8%)
PFS (months)				
Median (range)	9.76 (0.9, 52.5)	9.0 (1.8, 39.0)	5.3 (1.0, 22.0)	8.0 (0.9, 52.5)
12–24	10 (17.2%)	7 (15.9%)	4 (10.3%)	21 (14.9%)
25–36	2 (3.4%)	2 (4.5%)	0 (0%)	4 (2.8%)
> 36	2 (3.4%)	1 (2.3%)	0 (0%)	3 (2.1%)
< 12	42 (72.4%)	30 (68.2%)	31 (79.5%)	103 (73.0%)
Unknown	2 (3.4%)	4 (9.1%)	4 (10.3%)	10 (7.1%)

HER2 + : human epidermal growth factor receptor-positive; HR + : hormone receptor-positive; TN: triple negative; CT: chemotherapy; E: anthracycline; T: paclitaxel; ET: endocrine therapy; SERM: selective oestrogen receptor modulators; AI: aromatase inhibitor; RT: radiotherapy

**Table 2:** The status of metastasis and treatment response related with sampling

	HR + (N = 58)	HER2 + (N = 44)	TN (N = 39)	Overall (N = 141)
Metastasis sites				
Chest wall	18	8	8	34
Bone	35	20	10	65
Lymph node	36	25	27	88
Lung	20	15	15	50
Liver	16	16	10	42
Brain and others	22	6	10	35
Visceral metastasis				
No	28 (48.3%)	21 (47.7%)	18 (46.2%)	67 (47.5%)
Yes	30 (51.7%)	23 (52.3%)	21 (53.8%)	74 (52.5%)
No. of metastasis				
1	10 (17.2%)	16 (36.4%)	10 (25.6%)	36 (25.5%)
2	22 (37.9%)	16 (36.4%)	13 (33.3%)	51 (36.2%)
≥ 3	26 (44.8%)	11 (25.0%)	12 (30.8%)	49 (34.8%)
Unknown	0 (0%)	1 (2.3%)	4 (10.3%)	5 (3.5%)
Disease status of sampling				
First-line metastasis	43 (74.1%)	32 (72.7%)	29 (74.3%)	104 (73.8%)
Primary IV	15 (25.9%)	12 (27.3%)	6 (15.4%)	33 (23.4%)
Unknown	0 (0%)	0 (0%)	4 (10.3%)	4 (2.8%)
Best response after ctDNA sampling				
CR/PR	15 (25.9%)	22 (50.0%)	11 (28.2%)	48 (34.0%)
SD	33 (56.9%)	12 (27.3%)	16 (41.0%)	61 (43.3%)
PD	7 (12.1%)	6 (13.6%)	8 (20.5%)	21 (14.9%)
NE	3 (5.2%)	4 (9.1%)	4 (10.3%)	11 (7.8%)

HER2 + : human epidermal growth factor receptor-positive; HR + : hormone receptor-positive; TN: triple negative; AI: aromatase inhibitor; CR: complete response; PR: partial response; SD: stable disease; PD: progressive disease; NE: not evaluated

### Molecular profiling of Chinese advanced breast cancer

**Fig. 1:**
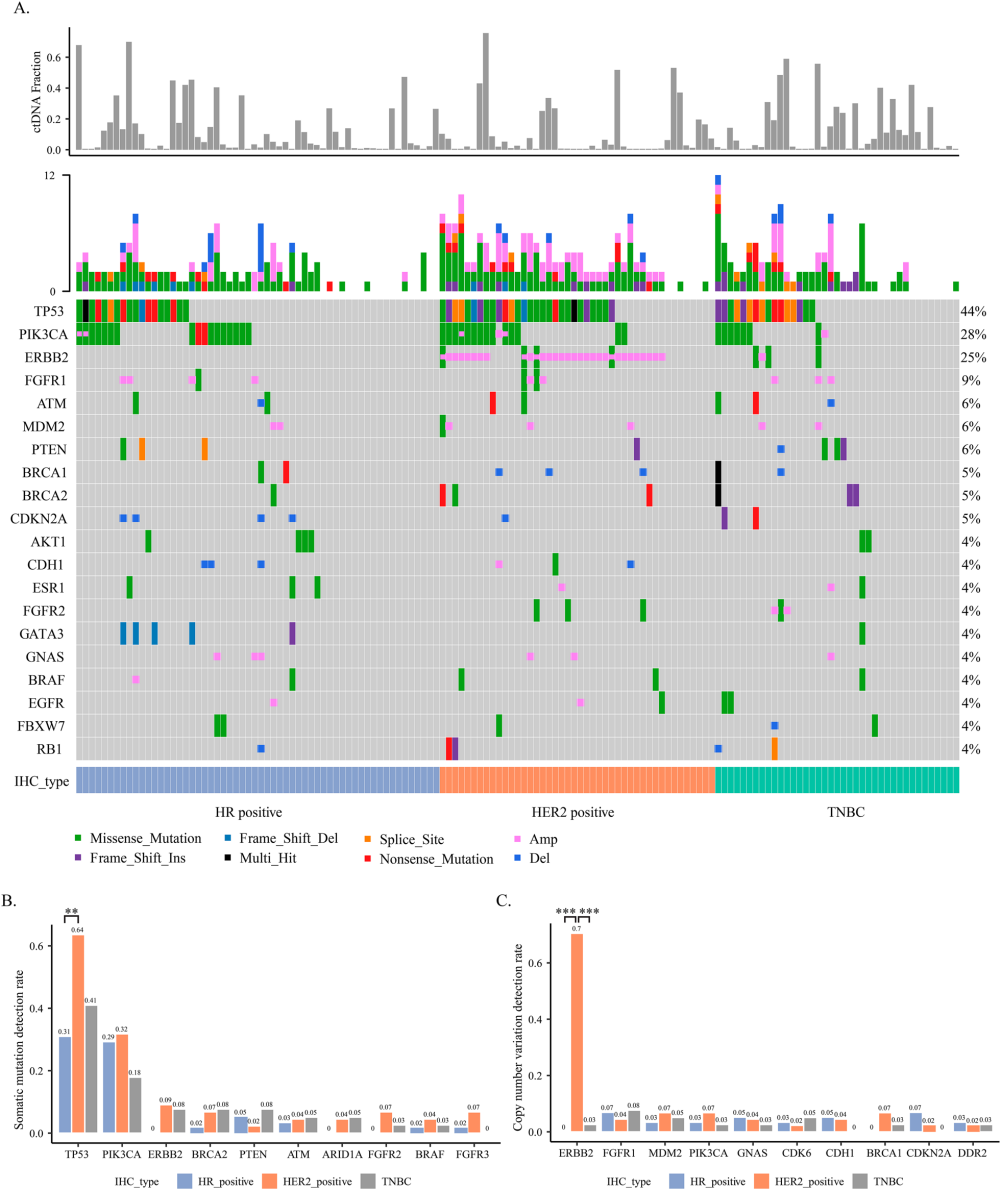
CtDNA mutation profile of Chinese ABC patients and the distribution of variations among different IHC subtypes. **A** Somatic mutation landscape of 141 Chinese ABC patients. The bar chart above the heatmap shows the ctDNA fractions of all baseline samples in the cohort. In the heatmap, the top bars depict the number of mutations a patient carried and the bars below denote different IHC subtypes. Each column represents one patient, and each row represents one gene. The text on the left represents gene names. The values on the right represent the mutation rates of these genes. Distributions of (**B**) the top 10 somatic mutations and (**C**) the top 10 CNVs among the three IHC subtypes. ABC, advanced breast cancer; ctDNA, circulating tumour DNA; IHC, immunohistochemistry; CNVs, copy number variations

Among the 141 analysed baseline plasma samples, 111 (78.7%) contained at least 1 somatic mutation, and 112 (79.4%) contained single nucleotide variations (SNVs) or CNVs. Collectively, 267 somatic alterations were detected across 65 genes, among which TP53 (44.0%), PIK3CA (28.4%), ERBB2 (24.8%), and FGFR1 (8.5%) were most frequently altered (Fig. 1A). ESR1 hotspot mutations (D538G and Y537S/N/C) were also detected in a subset of HR + patients at a lower frequency (4.0%). CNVs were detected in 32 genes, including amplifications of ERBB2 (22.7%), FGFR1 (6.4%), and PIK3CA (4.3%) and deletions of CDKN2A (3.5%), CDH1 (3.5%), and BRCA1 (2.8%) (Fig. 1A). In the heatmap show in Fig. 1A, each column represents one patient and each row represents one gene. The mutation rates are displayed on the right side of the heatmap. Next, the detection rates of somatic mutations and CNVs were compared among the different clinical subtypes (HR + , HER2 + , and TNBC). The results indicated that HER2 + patients had the highest frequency of TP53 alterations, which was significantly higher than that observed in HR + patients (64% vs. 31%, *P* < 0.009; Fig. 1B). For ERBB2 CNVs, amplifications occurred in 31/32 (96.9%) HER2 + patients. One amplification was observed in a TNBC patient, and no ERBB2 CNVs were detected in HR + patients (*P* < 0.001; Fig. 1C).

### Comparison of plasma ctDNA and tumour tissue sequencing results

**Fig. 2:**
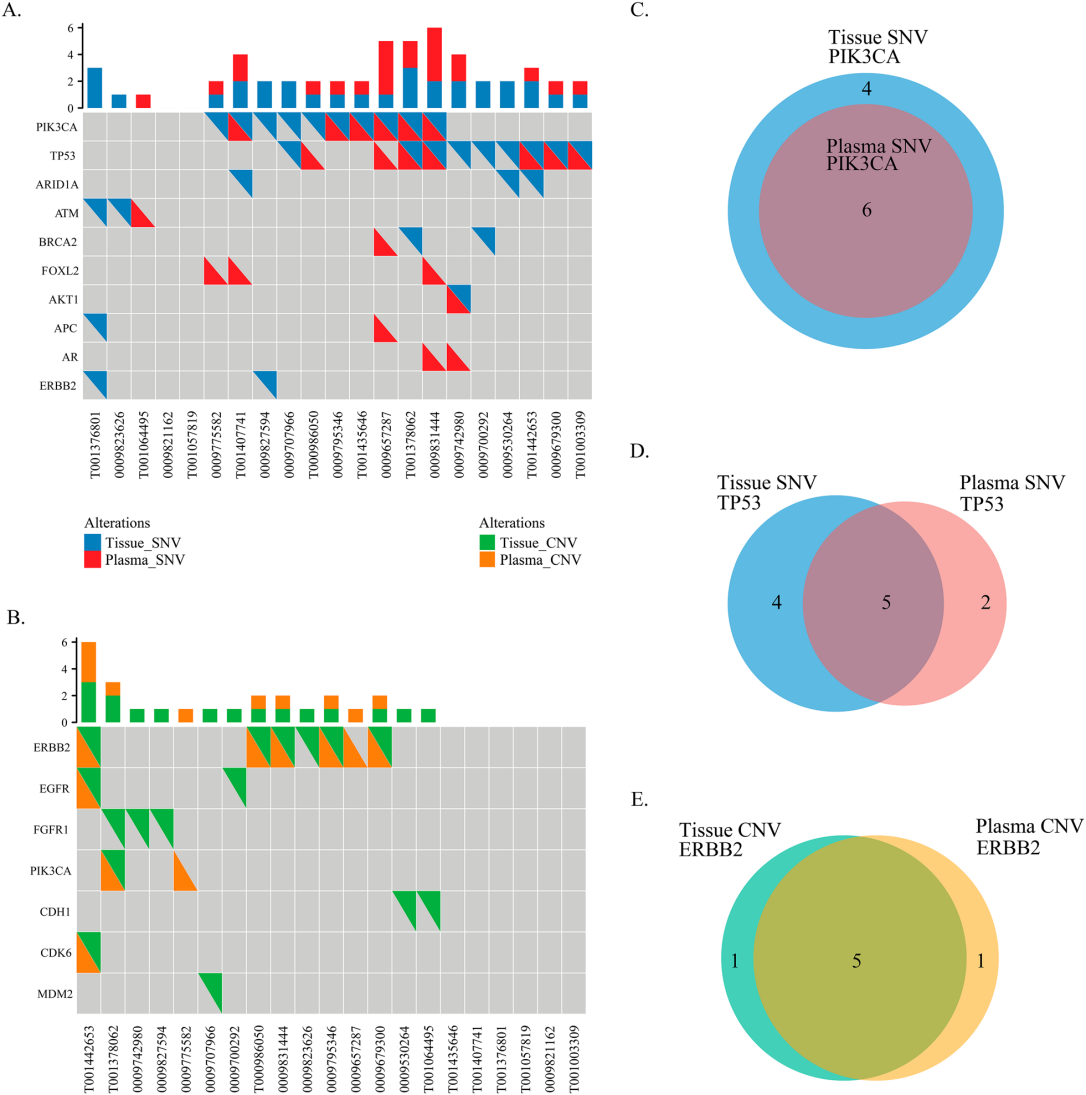
Concordance analysis of mutations between plasma and tissue samples. Concordance of (**A**) SNVs and (**B**) CNVs between tissue samples and plasma ctDNA. In the matrices, the top bar charts depict the number of mutations. The text below represents patient identities, and the text on the left represents gene names. Coloured triangles in the squares indicate mutations. If a square was filled with two coloured triangles, the same mutation was detected in the corresponding patient's plasma and tissue. Overlapping (**C**) PIK3CA SNVs, (**D**) TP53 SNVs, and (**E**) ERBB2 CNVs between tissue and plasma ctDNA. SNVs, single nucleotide variations; CNVs, copy number variations; ctDNA, circulating tumour DNA

Targeted NGS sequencing was applied to analyse plasma samples from 21 patients and their matching tumour tissues from metastatic sites. PIK3CA and TP53 were the two most common genes with SNVs, and ERBB2 was the most common gene with CNVs (Fig. 2A, B). In the matrices, each column represents one patient and each row represents one gene. A square filled with two coloured triangles suggests an identical mutation in tissue and plasma. Kappa tests were performed to test the consistency between the plasma samples and tissue samples. Cohen's kappa is a robust statistic used to compare the ability of different raters to classify subjects into one of several groups and can range from − 1 to + 1 [27]. A kappa value ≤ 0 indicates no agreement, 0.01–0.20 indicates no to slight agreement, 0.21–0.40 indicates fair agreement, 0.41–0.60 indicates moderate agreement, 0.61–0.80 indicates substantial agreement, and 0.81–1.00 indicates almost perfect agreement. PIK3CA SNVs were found in ten tissue samples and six plasma samples, with a match number of six (kappa = 0.61; Fig. 2C). Nine tissue samples and seven plasma samples were shown to have TP53 SNVs, and five pairs of samples had the same variants (kappa = 0.41; Fig. 2D). In addition, ERBB2 CNVs were detected in six tissue samples and six plasma samples, with a match number of five (kappa = 0.77; Fig. 2E).

### Genomic aberrations related to treatment response and prognosis

**Fig. 3:**
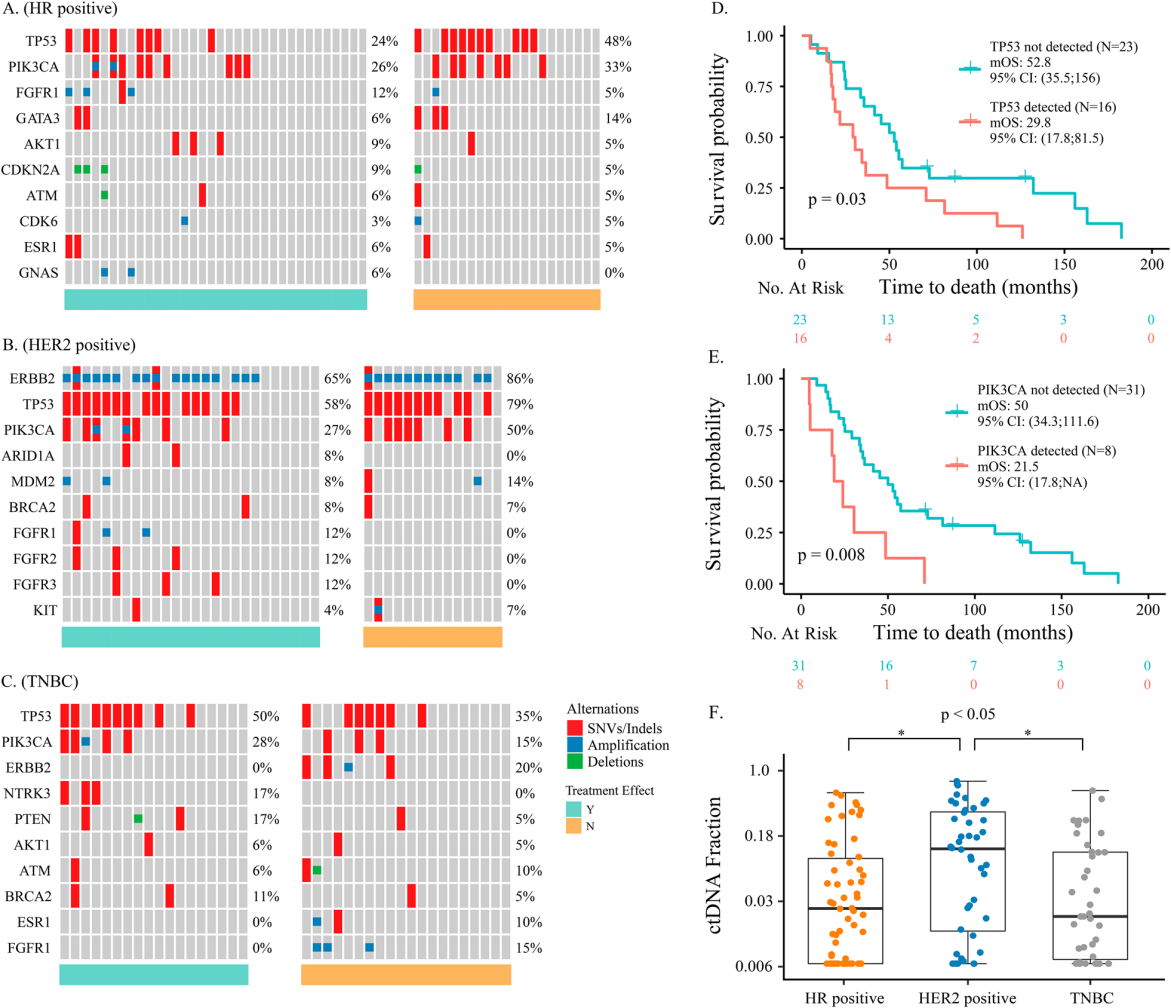
Oncoplots for drug-sensitive and drug-resistant (**A**) HR + samples, (**B**) HER2 + samples, and (**C**) TNBC samples. The green bars below the heatmap indicate samples collected from patients who are sensitive to treatment, while the orange bars indicate samples collected from patients who are resistant to treatment. Different colours of squares denote different types of mutations. Red represents SNVs/Indels, blue represents amplifications, and green represents deletions. Survival analyses of OS (**D**) between patients with or without TP53 mutations and (**E**) between patients with or without PIK3CA mutations. **F** Comparison of ctDNA fractions among the three IHC subtypes. The black lines represent the median of each group. HR + , hormone receptor-positive; HER2 + , human epidermal growth factor receptor 2-positive; TNBC, triple-negative breast cancer; SNVs, single nucleotide variations; Indels, insertions and deletions; OS, overall survival; ctDNA, circulating tumour DNA; IHC, immunohistochemistry

All patients were treated with current standard first-line therapy, and more than 50% of patients responded to the therapy. Correlation analysis was conducted to find particular variants that were related to the drug response for different subtypes of patients. The results indicated that the presence of PIK3CA/TP53 and FGFR1/2/3 variants was related to drug resistance in HR + patients (*P* = 0.05; Fig. 3A) and HER2 + patients (*P* = 0.07; Fig. 3B), respectively. However, no potential response-related somatic mutations or CNVs were found for TNBC patients (Fig. 3C). The green bars and the orange bars below the heatmap indicate samples collected from patients who were sensitive and resistant to treatment, respectively. SNVs/Indels, amplifications, and deletions are represented by red, blue, and green squares, respectively. In terms of outcomes, TNBC patients harbouring TP53 mutations (29.8 vs. 52.8 months, *P* = 0.03; Fig. 3D) and PIK3CA mutations (21.5 vs. 50 months, *P* = 0.008; Fig. 3E) had worse OS than those without corresponding mutations, but no significant difference in PFS or OS was found in HER2 + and HR + patients (data not shown).

### No significant difference in the ctDNA fraction between drug-sensitive and drug-resistant samples

As shown in Fig. 1A (the bar chart above the heatmap), the ctDNA fractions across all samples varied from 0.6% to 76%, with a median of 3.0%. HER2 + patients had a significantly higher ctDNA fraction (median, 13.0%) than HR + (median, 2.6%) and TNBC patients (median, 2.1%) (*P* < 0.02; Fig. 3F). To investigate whether the ctDNA fraction was associated with drug response, we compared the ctDNA fraction between drug-sensitive and drug-resistant patient samples among different subgroups. The results suggested that there was no significant difference in the ctDNA fraction between the two groups for all three subtypes (*P* > 0.05; Additional file 1: Fig. S1A).

### A high ctDNA fraction was associated with worse prognosis in HR + and TNBC patients

**Fig. 4:**
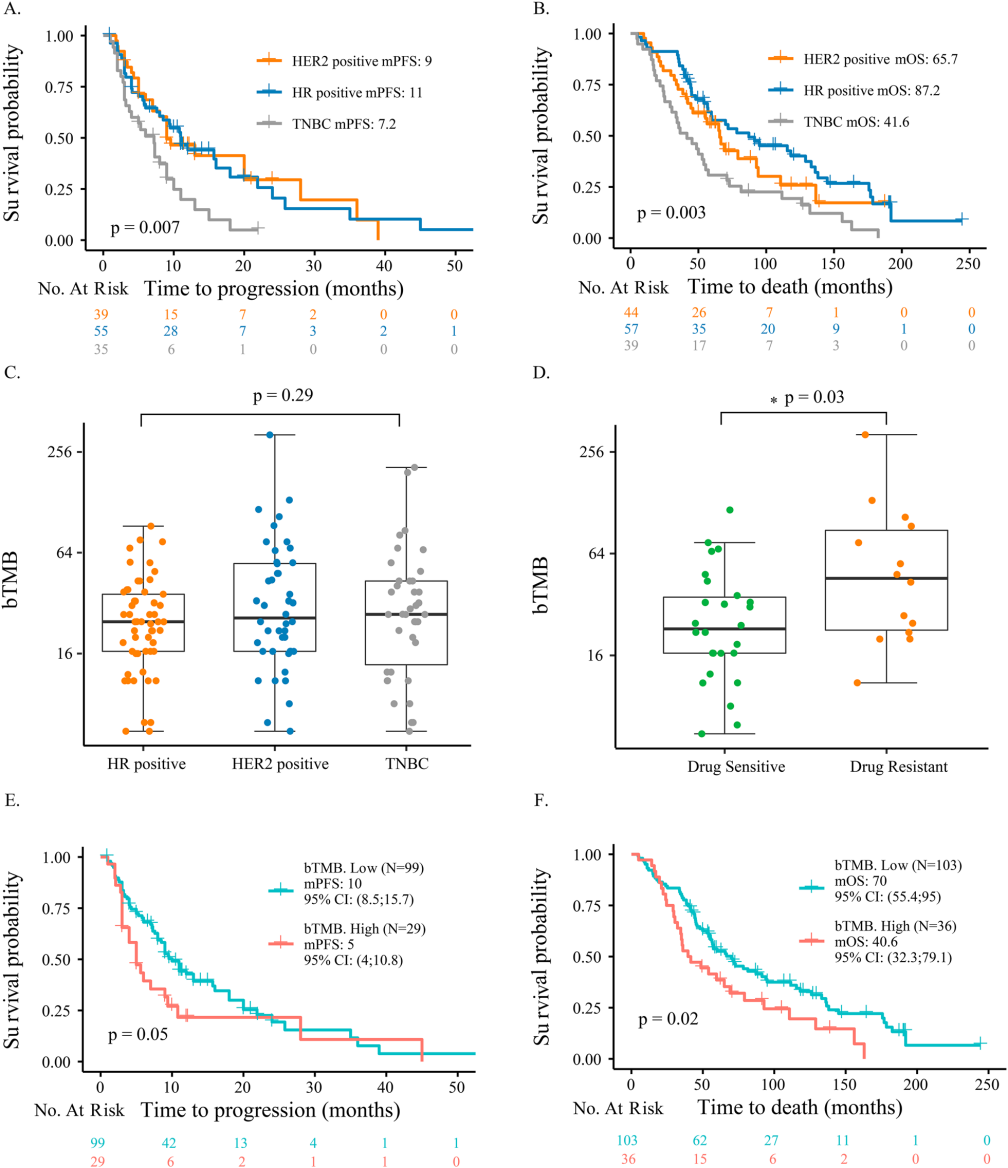
Survival analyses of (**A**) PFS and (**B**) OS among the three IHC subtypes. **C** Comparison of bTMB among the three IHC subtypes. **D** Comparison of bTMB between HER2 + drug-sensitive and drug-resistant samples. The black lines represent the median of each group. Survival analyses of (**E**) PFS and (**F**) OS between patients with low and high bTMB. PFS, progression-free survival; OS, overall survival; IHC, immunohistochemistry; bTMB, blood-based tumour mutation burden; HER2 + , human epidermal growth factor receptor 2-positive

**Fig. 5:**
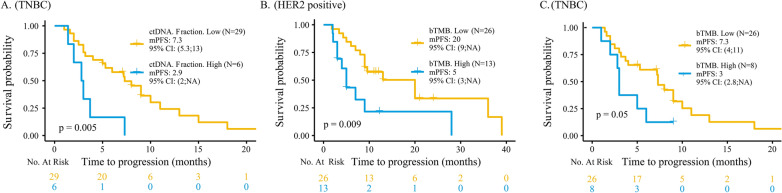
Survival analyses of PFS (**A**) between TNBC patients with low and high ctDNA fractions, (**B**) between HER2 + patients with low and high bTMB, and (**C**) between TNBC patients with low and high bTMB. PFS, progression-free survival; TNBC, triple-negative breast cancer; ctDNA, circulating tumour DNA; HER2 + , human epidermal growth factor receptor 2-positive; bTMB, blood-based tumour mutation burden

Survival analyses of the three clinical subtypes of patients revealed a significantly shorter PFS (7.2 vs. 9 vs. 11 months, *P* = 0.007; Fig. 4A) and OS (41.6 vs. 65.7 vs. 87.2 months, *P* = 0.003; Fig. 4B) for TNBC patients compared to the other two subtypes. All patients were dichotomized into two groups with high and low ctDNA fractions by the 75th percentile (0.174), as previously described [28–30]. A high ctDNA fraction was shown to be associated with a shorter PFS and OS for all subtypes, but these trends were not significant for most patients (*P* > 0.05; Additional file 1: Fig. S1B and Additional file 2: Fig. S2). One exception was that for TNBC patients, as a high ctDNA fraction was significantly associated with a shorter PFS in these patients (2.9 vs. 7.3 months, *P* = 0.005; Fig. 5A).

### bTMB was elevated significantly in drug-resistant HER2 + samples

The values of bTMB across all samples ranged from 0 to 324.0, with a median of 24.8. Comparison of bTMB among the different IHC subtypes revealed no significant differences (*P* = 0.29, Fig. 4C). We also explored the association between bTMB and drug response. In the HER2 + subtype, bTMB in samples collected from drug-resistant patients was significantly higher than that of samples collected from drug-sensitive patients (*P* = 0.03; Fig. 4D), but this association was not found in HR + and TNBC subtypes (Additional file 1: Fig. S1C).

### High bTMB was associated with worse prognosis in all patients

The patients were divided into high bTMB and low bTMB groups by the 75th percentile (43.3) as previously described [31–34]. Cox regression analysis revealed that patients with a high bTMB had a significantly shorter PFS (5 vs. 10 months, *P* = 0.05; Fig. 4E) and OS (40.6 vs. 70 months, *P* = 0.02; Fig. 4F) than those with a low bTMB. For different subtypes, HER2 + (5 vs. 20 months, *P* = 0.009; Fig. 5B) and TNBC patients (3 vs. 7.3 months, *P* = 0.05; Fig. 5C) with a high bTMB had a significantly shorter PFS than those with a low bTMB.

### Disease monitoring with ctDNA sequencing

Plasma samples were collected at disease progression from 31 out of the 141 patients and were analysed to monitor genomic variants during clinical progression. Compared with the corresponding baseline samples, several acquired variants were detected in samples collected from HR + and TNBC patients at progression. These variants included mutations in TP53, PIK3CA, and ESR1, the deletion of CDKN2A, and the amplification of FGFR1. In contrast, some variants detected at baseline in HER2 + patients, such as TP53 and PIK3CA mutations, were not detected at disease progression (Additional file 3: Fig. S3).

## Discussion

In recent years, efforts have been made to investigate the genomic complexity of breast cancer [2, 35–38]. However, these studies were either retrospective or lacked ctDNA-based dynamic disease monitoring and clinical management. Breast cancer is a heterogeneous disease that varies across different clinical subtypes, populations and geographical regions, all of which exhibit variable therapeutic responses [39, 40]. With the increasing incidence rate of breast cancer worldwide, it is critical to identify region-specific genomic variants and relevant therapeutic targets. In this prospective study, a novel 152-gene targeted NGS panel was used to evaluate the mutation landscape of ctDNA collected from 141 Chinese ABC patients. In addition, we profiled drug response-related genomic variants, evaluated the prognostic value of the ctDNA fraction and bTMB, and assessed the ability of ctDNA to monitor disease progression.

In this study, molecular profiling of ABC revealed genomic variants consistent with several previous reports, and TP53 (44.0%) and PIK3CA (28.4%) were the two most commonly altered genes [2, 35, 38, 41, 42]. TP53 is a well-characterized tumour suppressor gene that acts as a regulator of key cellular processes involved in controlling cell proliferation and maintaining genomic stability [43, 44]. Almost all hallmark features of cancer are impacted by the functions of the TP53 protein and correlate with genomic alterations in TP53 pathways [45]. In other words, the occurrence of many tumours requires TP53 mutations as a prerequisite. Indeed, TP53 was shown to be frequently altered in previous sequencing studies, with frequencies ranging from 38.24 to 74.11% [2, 35, 38, 41, 42, 46]. In particular, the mutation rates of TP53 were 38.24%, 41.67%, 64.1%, 43.27%, and 47.0% in Chinese populations [2, 35, 41, 42, 46]. Therefore, it is not surprising to find a high mutation rate of TP53 in this study. However, in regard to individual situations, more efforts are needed to reveal the specific role of TP53. HER2 + patients showed a significantly higher TP53 mutation rate than HR + patients. TNBC patients harbouring TP53 and PIK3CA mutations showed a significantly longer OS than those without these alterations. There is clear evidence that TP53 is associated with poor prognosis of HR + disease, while its clinical significance in HER2 + and TNBC patients remains controversial [47]. Considering the high degree of heterogeneity of TP53 mutations, further large-scale sequencing studies are needed to address its clinical impact and association with response to therapy in distinct subtypes. PIK3CA is the most frequently mutated oncogene in human cancers [48, 49]. The continuous activation of AKT that is induced by PIK3CA mutations can promote the growth and transformation of mammary epithelial cells and may explain the high mutation rate of PIK3CA [50]. The approval of alpelisib has rendered the detection of PIK3CA mutations clinically actionable, as everolimus, an mTOR inhibitor, does not directly target PIK3CA mutations. Thus, HR + /HER2- ABC patients harbouring PIK3CA mutations can be offered another targeted therapy choice after endocrine resistance occurs [51]. For TNBC, the prognostic impact of PIK3CA mutations remains debatable, and evidence for the clinical application of PIK3CA inhibitors is still lacking [52, 53]. The BELLE-4 study tested the efficacy of buparlisib (a pan-PIK3CA inhibitor) in combination with placebo or paclitaxel in HER2- ABC patients [54]. Approximately 25% of the patients were TNBC patients who showed a worse outcome with the addition of burpalisib than placebo (5.2 vs. 9.3 months; hazard ratio 1.86, 95% CI 0.91–3.79). Nonetheless, combination approaches targeting PIK3CA with other targeted drugs, such as androgen receptor and CDK4/6 inhibitors, may provide a potential therapeutic direction for specific subsets of TNBC patients [53]. Several ESR1 hotspot mutations within the ligand-binding domain, including D538G and Y537S/N/C, were also detected at a lower frequency (4%). Given established treatment-associated patterns of acquired mutations in ESR1, it is not surprising that the mutation rate was not high in the setting of first-line treatment [55].

ERBB2 was the gene with the most frequent occurrence of CNVs and had an amplification rate of 22.7% (32/141) in the entire cohort and 70.5% (31/44) in the HER2 + population. Compared with the study by Davis et al., the amplification rate in ERBB2 was higher in our cohort (70.5% vs. 44.0%) [38]. This difference may be explained by two factors. The first factor is the patient population. We enrolled Chinese patients in this study, while the study by Davis et al. was conducted in the United States. This explanation is supported by a previous study by Xiao et al. which also revealed a high amplification rate of ERBB2 (90.3%, 158/175) in HER2 + Chinese patients [46]. We (PredicineCARE, developed by Huidu Shanghai Medical Sciences Ltd.) and Davis et al. (Guardant360 assay, Guardant Health, Inc., Redwood City, CA) used different NGS panels to detect variants. The higher detection rate may indicate the improved ability of copy number detection from our NGS assay. Notably, one TNBC patient presented with an ERBB2 CNV, indicating tumour heterogeneity and the potential need to reassess HER2 status by ctDNA during the clinical management of TNBC [56]. These findings underscore the feasibility of tracing ERBB2 CNVs with liquid biopsy during disease progression and introduce the possibility for applying anti-HER2 therapy. In the analysis of drug response-related variants, the detection of individual SNVs or CNVs showed no correlation with drug response. However, the combination analysis of SNVs with CNVs revealed potential associations between the PIK3CA/TP53 and FGFR1/2/3 variants and drug resistance in HR + and HER2 + patients, respectively. These findings suggest that integrating multiple genetic alterations could improve the identification of treatment resistance-related mechanisms compared to measuring a single alteration [57].

CtDNA levels can dynamically reflect the tumour burden of a patient and predict disease progression prior to imaging [58]. Moreover, several studies have revealed the prognostic role of the ctDNA fraction in ABC patients [58–60]. Stover et al. found that a ctDNA fraction of ≥ 10% correlated with a worse metastatic OS (6.4 vs. 15.9 months) [59], while a cut-off of 0.5% ctDNA (MAF) was regarded as prognostic for both PFS and OS in another report [58]. In the present study, a cut-off of 0.174 was used to discriminate patients with high or low ctDNA fractions. Based on this grouping, we found a significant difference in PFS between TNBC patients with high vs. low ctDNA fractions (2.9 vs. 7.3 months, *P* = 0.005). However, no significant differences in PFS were observed in other subtypes. This could result from subject variations in disease onset, diagnosis, or intervention since this study contained all subtypes of patients and multiple treatment regimens. Moreover, this heterogeneity could result in opposite associations between the ctDNA fraction and prognosis among different subtypes. In addition to its prognostic role, the ctDNA fraction can reflect the panel sensitivity, as the panel sensitivity decreased if samples displayed low tumour fractions. Therefore, we evaluated the correlation between the ctDNA fraction and the number of mutations in all baseline plasma samples using the Pearson correlation coefficient, and the results suggested a significant positive correlation (R = 0.56, *P* < 0.05; Additional file 4: Fig. S4A). The number of mutations in samples with ctDNA fractions of 0–1% was significantly lower than that in samples with ctDNA fractions of 1–5% and > 5% (Additional file 4: Fig. S4B). These findings also illustrate the importance of improving sequencing sensitivity.

TMB is a measure of the somatic mutation frequency and mutation accumulation process of a tumour. In tumorigenesis, some nondriver mutations will lead to the activation of an antitumour response by generating neoantigens that are recognized by the immune system [61]. Consequently, TMB is regarded as a biomarker that is associated with the response to immune checkpoint inhibitors in several types of cancer [17]. Despite being a promising pancaner tool, the use of TMB is limited by several key remaining issues, including variable clinical impacts across cancer types and the lack of a standardized cut-off value [17]. In this study, a cut-off of 43.3 was adopted to define high vs. low bTMB, and patients with high bTMB showed a shorter PFS (5 vs. 10 months, *P* = 0.05) and OS (40.6 vs. 70 months, *P* = 0.02) than those with low bTMB. These findings are consistent with previous reports that suggested that TMB was a prognostic factor for poor outcome [19, 20]. Even so, the association between bTMB and prognosis should be interpreted with caution because of differences at the individual level, which may reduce its usability in practical situations. Indeed, in our further analyses based on subtypes, these associations were significant only in HER2 + (PFS 5 vs. 20 months, *P* = 0.009) and TNBC patients (PFS 3 vs. 7.3 months, *P* = 0.05). Hence, more research investigating the clinical utility of ctDNA-derived biomarkers in ABC is needed to address this issue. Interestingly, HER2 + samples displayed significantly elevated bTMB. Since recent evidence has indicated elevated programmed cell death-ligand 1 and tumour infiltrating lymphocyte expression in HER2 + disease [62], further studies exploring the possibility of implementing immunotherapy in such patients are warranted.

Previous sequencing studies have revealed the concordant detection of DNA variants across matched sets of ctDNA and tumour tissue [10–14]. Davis et al. described the genomic landscape of ctDNA in 255 ABC patients. They found an agreement of 79–91% between 105 pairs of ctDNA and tissue, with alterations in PIK3CA and TP53 showing moderate concordance (kappa = 0.5513 and 0.5809, respectively) [38]. In this study, we also found moderate concordance for the SNVs in PIK3CA and TP53 (kappa = 0.61 and 0.41, respectively). In addition, a high concordance was revealed for ERBB2 CNVs (kappa = 0.77), which was consistent with a report by Zhou et al. [35]. These findings suggest the equivalent utility of ctDNA with tissue sequencing in developing targeted therapeutic approaches for HER2 + patients.

We also performed longitudinal monitoring of disease progression to observe the dynamic changes in gene mutations and amplifications in ctDNA. The genomic variants detected in ctDNA at baseline and at disease progression were compared. Overall, the plasma ctDNA from samples collected at the time of clinical progression in HR + and TNBC patients had acquired genomic variants, indicating clonal and subclonal responses to treatment. In contrast, genomic variants detected at baseline in HER2 + patients were not detected at progression. In HR + patients, newly acquired ESR1 mutations at the time of disease progression were observed in four patients, which is consistent with previous findings that the emergence of ESR1 mutations is associated with the development of endocrine resistance [55]. Other emerging genomic variants included FGFR amplification and CDKN2A deletion events. Aberrant FGFR signalling has been identified as a mechanism that drives tumour growth and promotes angiogenesis [63]. The amplification of FGFR, which occurs in approximately 10–16% of HR + patients [60], has been shown to mediate endocrine resistance [64]. CDKN2A (cyclin-dependent kinase inhibitor 2A) is a tumour suppressor gene that was first reported in 1993, and it is negatively regulated by the CDK4/6/RB pathway [65]. Despite rare alterations of this gene in breast cancer (approximately 5.8%) [66], CDKN2A deletions were reported to be associated with poor outcomes in luminal B ER + patients [67]. Importantly, the deletion of this gene implicates CDK4/6 as a therapeutic target to some extent [68]. Taken together, the acquired FGFR amplification and CDKN2A deletion variants in progression samples may explain the disease progression in these HR + patients.

There are several limitations that should be mentioned. First, the sample size was relatively small, and only a subset of patients had matched blood and tissue samples. These factors may decrease the concordance of detected genomic variants. Second, the samples were collected in a single centre and sequenced by a single NGS assay. Hence, the results from this study need to be validated in other research centres using distinct sequencing assays. Third, since the NGS panel applied in this study only included 152 genes, its ability to detect tumour burden is theoretically inferior to genome-wide tests. Thus, the association between bTMB and prognosis needs more verification. Fourth, the sensitivity of this panel may decrease if samples display a lower ctDNA fraction, and the ctDNA fraction of all samples in this study varied greatly (Additional file 5). Finally, although treatment response-related variants were analysed, the effects of drug interventions guided by ctDNA profiling were not studied. Further studies will be required to evaluate the impact of ctDNA profiling-guided drug interventions on patient outcomes to establish the clinical utility of NGS liquid biopsy in the management of ABC patients.

In conclusion, this prospective study profiled the mutation landscape of Chinese ABC patients who underwent first-line standard treatment and demonstrated the clinical validity of ctDNA-based genomic analysis. Further studies are warranted to investigate the relationship between drug interventions and genomic changes.

## Supplementary Information

**Additional file 1. Figure S1**. (A) Comparisons of ctDNA fractions between drug-sensitive and drug-resistant patients. (B) Survival analyses between all patients with low and high ctDNA fractions. (C) Comparisons of bTMB between drug-sensitive and drug-resistant samples. The black lines represent the median of each group. ctDNA, circulating tumour DNA; bTMB, blood-based tumour mutation burden.

**Additional file 2.Figure S2**. Survival analyses between (A) HR + patients, (B) HER2 + patients, and (C) TNBC patients with low and high ctDNA fractions. HR + , hormone receptor-positive; HER2 + , human epidermal growth factor receptor 2-positive; TNBC, triple-negative breast cancer; ctDNA, circulating tumour DNA.

**Additional file 3.Figure S3**. Survival analyses between (A) HR + patients, (B) HER2 + patients, and (C) TNBC patients with low and high bTMB. (D) Comparison of mutations between plasma samples at baseline and disease progression. In the heatmap, the top bars depict the total number of mutations a patient carried at baseline and at disease progression. The bars below denote different IHC subtypes. Each column represents one patient. Every two rows represent the status of one gene at baseline (upper) and at disease progression (lower). The text on the left represents gene names and sampling time points. The values on the right represent the mutation rates of these genes. Different colours of squares denote different types of mutations. Red represents SNVs/Indels, blue represents amplifications, and green represents deletions. HR + , hormone receptor-positive; HER2 + , human epidermal growth factor receptor 2-positive; TNBC, triple-negative breast cancer; bTMB, blood-based tumour mutation burden; IHC, immunohistochemistry; SNVs, single nucleotide variations; Indels, insertions and deletions.

**Additional file 4.** **Figure S4**. Correlation between the ctDNA fraction and number of mutations. (A) Pearson correlation coefficient between the ctDNA fraction and number of mutations in 141 baseline plasma samples. The X-axis represents the ctDNA fraction, and the Y-axis represents the number of mutations. The blue slash indicates the correlation trend between the two sets of data. (B) Comparison of the number of mutations among samples with different ctDNA fractions. The X-axis represents the three groups divided according to ctDNA fraction and the Y-axis represents the number of mutations.

**Additional file 5**. Original sequencing data including IHC type of all patients, SNVs, CNVs, and ctDNA fraction and bTMB of baseline plasma, SNVs and CNVs of plasma samples used for disease monitoring, and SNVs and CNVs of tissue samples. IHC, immunohistochemistry; SNVs, single nucleotide variations; CNVs, copy number variations; bTMB, blood-based tumor mutation burden.

## Data Availability

All data and materials supporting the conclusions of this article were available in the figures, tables, and supplementary materials, which are available to authorized users.

## Abbreviations

ABC: Advanced breast cancer
cfDNA: Circulating cell-free DNA
ctDNA: Circulating tumor DNA
NGS: Next generation sequencing
bTMB: Blood-based tumor mutation burden
HR +: Hormone receptor-positive
HER2 +: Human epidermal growth factor receptor 2-positive
TNBC: Triple-negative breast cancer
PKUCH: Peking University Cancer Hospital
ER: Oestrogen receptor
PR: Progesterone receptor
PFS: Progression-free survival
OS: Overall survival
IHC: Immunohistochemistry
FISH: Fluorescence in situ hybridization
SNVs: Single nucleotide variations
CNVs: Copy number variations
Indels: Insertions and deletions
MAF: Mutation allele frequency
CIs: Confidence intervals
